# Whole-Genome Analysis of *bla*_NDM_-Bearing *Proteus mirabilis* Isolates and *mcr-1*-Positive *Escherichia coli* Isolates Carrying *bla*_NDM_ from the Same Fresh Vegetables in China

**DOI:** 10.3390/foods12030492

**Published:** 2023-01-20

**Authors:** Chang-An Li, Cai-Hong Guo, Ting-Yu Yang, Fang-Yu Li, Feng-Jing Song, Bao-Tao Liu

**Affiliations:** 1 College of Veterinary Medicine, Qingdao Agricultural University, Qingdao 266109, China; 2 Institute of Plant Protection, Qingdao Academy of Agricultural Sciences, Qingdao 266100, China

**Keywords:** *carbapenemase genes*, *mcr-1*, concurrence, *P. mirabilis*, *E. coli*

## Abstract

The global spread of colistin or carbapenem-resistant Enterobacteriaceae (CRE) has been a pressing threat to public health. Members of Enterobacteriaceae, especially *Proteus mirabilis* and *Escherichia coli,* have been prevalent foodborne pathogens and such pathogens from fresh vegetables have triggered foodborne illness in China. However, reports about CRE, especially *P. mirabilis* from fresh vegetables, are still lacking. In this study, we identified five *bla*_NDM_-positive *P. mirabilis* and five *bla*_NDM_-positive generic *E. coli* concurrently from five fresh vegetables in two markets from China, and four of the five *E. coli* also carried *mcr-1.* The 10 isolates were characterized with methods including antimicrobial susceptibility testing, conjugation, whole-genome sequencing and phylogenetic analysis. All 10 isolates were multidrug-resistant (MDR). *bla*_NDM-5_ in five *E. coli* isolates and one *P. mirabilis* carrying *bla*_NDM-5_ was located on similarly transferable IncX3 plasmids, while transferably untypable plasmids were the carriers of *bla*_NDM-1_ in four *P. mirabilis* isolates from different types of vegetables/markets. *mcr-1* in the four *bla*_NDM_-_5_-positive *E. coli* was located on similarly non-conjugative IncHI2 MDR plasmids lacking transfer region. Notably, IS*CR1* complex class 1 integron capable of capturing *bla*_NDM-1_ was found on all untypable plasmids from *P. mirabilis*, and five copies of IS*CR1* complex class 1 integron containing *bla*_NDM-1_ even occurred in one *P. mirabilis*, which showed high-level carbapenem resistance. Plasmid and phylogenetic analysis revealed that the *bla*_NDM_-positive *P. mirabilis* and *E. coli* from fresh vegetables might be derived from animals and transmitted to humans via the food chain. The concurrence of *bla*_NDM_-positive *P. mirabilis* and *E. coli* carrying both *mcr-1* and *bla*_NDM_ in different types of fresh vegetables eaten raw is alarming and threatens food safety. Sustained surveillance of these foodborne pathogens among fresh vegetables is urgent to ensure the health of food consumers. We report for the first time the concurrence of *bla*_NDM_-positive *P. mirabilis* and *mcr-1*-bearing *E. coli* carrying *bla*_NDM_ from the same fresh vegetables.

## 1. Introduction

The global spread of carbapenem-resistant Enterobacteriaceae (CRE) has been a pressing threat to public health [1], and such pathogens have been disseminated widely in clinical settings in many counties, including China [2]. New Delhi metallo-β-lactamase (NDM) has been the main type of carbapenemases conferring resistance to almost all β-lactams, and CRE is the most common NDM carriers [3]. NDM-producing CRE isolates have been frequently found in humans [4], hospital wastewater [5] and animals [3] around the world. With the rapid increase in CRE, colistin has been re-used as the “last line of defense” for the treatment of CRE [6]. However, several *mcr* variants have been identified in various Enterobacteriaceae species [7], challenging the efficacy of colistin. The concurrence of colistin resistance in CRE isolates has been a great clinical concern, challenging the clinical usefulness of both colistin and carbapenems. In recent years, *mcr* has emerged in CRE isolates from humans [8], animals [9] and retail meats [10] in many countries.

Fresh vegetables have been associated with foodborne diseases [11], and the bacteria contaminated in fresh vegetables often serve as a reservoir of antimicrobial resistance genes [12,13]. The consumption of fresh vegetables eaten raw may result in transmitting antimicrobial resistant bacteria to humans [14], posing a threat to the health of consumers. The *mcr*-positive isolates among fresh vegetables have been reported in a few countries, including Portugal [15], Switzerland [16], South Korea [17] and China [18], and the prevalence of such bacteria in vegetables remains very low. Worryingly, CRE has also been found in vegetables in limited reports with a very low prevalence [14,19,20]. All these findings suggest that carbapenem resistance and colistin resistance have emerged in fresh vegetables, and one of the major concerns is the concurrence of carbapenemase genes and *mcr* in the same isolate from fresh vegetables eaten raw. To date, two *E. coli* isolates carrying both *mcr-1* and *bla*_NDM_ in fresh vegetables have been only reported in our previous study [12]. Sustained surveillance of CRE carrying *mcr* among fresh vegetables is needed to ensure food consumers’ health.

*Proteus mirabilis* is intrinsically resistant to polymyxins and tigecycline and is a zoonotic opportunistic pathogen that belongs to Enterobacteriaceae. *P. mirabilis* is notorious for its ability to actively disseminate antimicrobial resistance genes through horizontal gene transfer, and multidrug-resistant (MDR) *P. mirabilis* isolates, including some carrying *bla*_NDM_, have triggered nosocomial infections in many counties [21]. Furthermore, *P. mirabilis* is generally associated with food spoilage and can also cause foodborne illness when consumed in contaminated food, especially meat and vegetables [22,23]. *P. mirabilis* from vegetables has been linked with foodborne illness [24]. However, there has been no report about *bla*_NDM_-positive *P. mirabilis* isolates from fresh vegetables, especially in China, in which CRE isolates from vegetables have occurred. To gain insight into the characteristics and potential role of foodborne CRE isolates in disseminating MDR to humans, the present study was carried out to identify carbapenem-resistant *P. mirabilis* and *mcr-1*-positive *E. coli* possessing *bla*_NDM_ in fresh vegetables. The molecular and sequence characteristics of these foodborne CRE isolates were further scrutinized by using bioinformatic analysis.

## 2. Materials and Methods

### 2.1. Identification of P. mirabilis and E. coli Harboring Carbapenemases Genes

A total of 720 fresh vegetable samples were included in this study, including 712 samples in our previous report [12] and eight fresh vegetable samples (one green pepper, three cucumber, two lettuce and two tomato samples) collected in one farmer’s market and one supermarket in Hangzhou of Zhejiang Province, China, in June 2019. These samples were processed with MH broth (Hope Bio-Technology Co., Qingdao, China) containing meropenem (1 µg/mL) and vancomycin (30 µg/mL) using the same protocol as previously described [20]. Carbapenem-resistant *P. mirabilis* isolates were isolated by streaking onto *Salmonella Shigella* (SS) agar plates (Hope, Qingdao, China) containing 30 µg/mL of vancomycin and 1 µg/mL of meropenem and incubated at 37 °C for 16–20 h. The representative clones were isolated, purified and confirmed to be *P. mirabilis* by using 16 S rDNA sequencing [25]. The samples containing carbapenem-resistant *P. mirabilis* were subsequently used to isolate *E. coli* using MacConkey Agar (Hope, Qingdao, China) supplemented with meropenem (1 µg/mL), as we previously reported [12]. The obtained meropenem-resistant *P. mirabilis* and *E. coli* isolates were detected for the carbapenemase genes (*bla*_NDM_, *bla*_KPC_, *bla*_IMP_, *bla*_OXA-48_, *bla*_VIM_, *bla*_GIM_, *bla*_SPM_, *bla*_DIM_, *bla*_AIM_, *bla*_BIC_ and *bla*_SIM_) using the previously described method [26]. The *mcr* (*mcr-1*~*mcr-10*) genes were also detected among the *E. coli* isolates [7,27].

### 2.2. Detection of Virulence Genes for P. mirabilis and Diarrheagenic E. coli

Diarrheagenic *E. coli* pathotypes, including enteropathogenic *E. coli* (EPEC), enterotoxigenic *E. coli* (ETEC), shiga toxin-producing *E. coli* (STEC), enteroinvasive *E. coli* (EIEC) and enteroaggregative *E. coli* (EAEC) were identified using the previously described PCR method [28]. The criteria used for determining pathotypes were as follows: isolates carrying *eaeA* and *escV* and possible additional genes *ent* and *bfpB* were EPEC; isolates carrying *elt* and/or *estla* or *estlb* were ETEC; isolates carrying *stx1* and/or *stx2* and possible additional *eaeA* were STEC; isolates carrying *invE* and *ipaH* were EIEC; isolates carrying *pic* and/or *aggR* were EAEC. Diffusely adherent *E. coli* (DAEC) was identified by specific PCR for *afa/dr* as previously reported [29]. For *P. mirabilis*, the detection of eight virulence genes (*ptA*, *zapA*, *ucaA*, *ireA*, *hpmA*, *mrpA*, *pmfA* and *atfA*) that are often found in isolates from urinary tract infection was performed by using PCR, as previously described [30].

### 2.3. Antimicrobial Susceptibilities

Antimicrobial susceptibilities to 13 antimicrobials were determined for the obtained meropenem-resistant *E. coli* isolates using the broth microdilution method [31]. The 13 antimicrobial agents included cefotaxime, ceftazidime, ampicillin, meropenem, ciprofloxacin, levofloxacin, nalidixic acid, kanamycin, amikacin, streptomycin, tigecycline, tetracycline and colistin. Except for tigecycline and colistin, the susceptibilities to the remaining 11 antimicrobials were also measured for *P. mirabilis* isolates using the broth microdilution method. The resistant criteria recommended by the 2019 EUCAST were used for tigecycline and colistin [32], and the results for the remaining 11 drugs were interpreted according to the CLSI breakpoints [31].

### 2.4. Multilocus Sequence Typing of E. coli

Multilocus sequence typing (MLST) of *E. coli* carrying carbapenemases genes was performed [33]. Sequence types of *E. coli* were identified according to the databases (https://pubmlst.org/bigsdb?db=pubmlst_escherichia_seqdef&page=sequenceQuery (accessed on 1 June 2022)) and (https://pubmlst.org/bigsdb?db=pubmlst_escherichia_seqdef&page=profiles (accessed on 1 June 2022)).

### 2.5. Plasmid Conjugation and Replicon Typing

To investigate the transferability of *bla*_NDM_ and *mcr-1*, conjugation experiment was carried out using *P. mirabilis* isolates harboring carbapenemase genes and *mcr*-positive *E. coli* carrying carbapenemase genes as the donors. *E. coli* C600 resistant to streptomycin was used as a recipient, and the broth mating method was performed as previously reported [34]. Eosin methylene blue (EMB) agar containing both colistin (2.5 µg/mL) and streptomycin (2000 µg/mL) was used to screen *mcr*-positive transconjugants, while MacConkey agar plates containing both meropenem (1 µg/mL) and streptomycin (2000 µg/mL) were used to isolate transconjugants harboring carbapenemase genes. Transconjugants were confirmed using the PCRs mentioned above, and antimicrobial susceptibilities for transconjugants were also determined. Plasmid replicon types within transconjugants were detected using a PCR method [35], and the IncI2 and IncX3 replicons were also screened [36,37].

### 2.6. Whole Genome Sequencing and Phylogenetic Analysis

To investigate the genetic properties of isolates and plasmids carrying *mcr*/*bla*_NDM_, whole genome sequencing (WGS) was carried out for the meropenem-resistant *P. mirabilis* (*n* = 2), *E. coli* (*n* = 3) and the transconjugants (*n* = 3) from *P. mirabilis*. Briefly, the total DNA of these isolates was extracted, respectively, and then was subjected to 250 bp paired-end WGS using the Illumina Hiseq 2500 platform (Illumina, Santiago, CA, USA). SPAdes v3.8.2 was used to assemble the Illumina sequence reads. Oxford Nanopore MinION sequencer was used to further sequence the *E. coli* isolate M15061H and *P. mirabilis* M15061B from the same lettuce sample. Unicycler v0.4.7 was used to assemble the MinION reads, and high-quality Illumina reads. MLSTs of *E. coli* isolates were confirmed by submitting genome sequences to MLST 2.0 (https://cge.cbs.dtu.dk/services/MLST/ (accessed on 1 July 2022)) [38]. Genome sequences were then subjected to Resfinder 3.1 (https://cge.cbs.dtu.dk/services/ResFinder/ (accessed on 1 July 2022)) and PlasmidFinder 2.0 (https://cge.cbs.dtu.dk/services/PlasmidFinder/ (accessed on 1 July 2022)) to obtain the antimicrobial resistance genes and plasmid replicon types, respectively. VirulenceFinder 2.0 (https://cge.food.dtu.dk/services/VirulenceFinder/ (accessed on 1 July 2022)) was used to analyze the virulence genes in the genome sequences of *E. coli*. The obtained genomes were annotated using the NCBI Prokaryotic Genome Annotation Pipeline (PGAP) server.

To analyze the phylogenetic relationships of the *mcr-1*-positive *E. coli* isolates carrying *bla*_NDM_ in this study and those reported from humans and animals, we used our 3 *E. coli* genomes from fresh vegetables and 57 assembled genomes carrying *mcr-1* or *bla*_NDM_ from different countries and sources in the pathogen database of NCBI (https://www.ncbi.nlm.nih.gov/pathogens (accessed on 15 July 2022)) (Table S1). Furthermore, the 2 *bla*_NDM_-positive *P. mirabilis* (M15061B and M15101B) from fresh lettuces in our study and 15 genomes of *bla*_NDM_-positive *P. mirabilis* from the NCBI database (1 from humans in Italy, 2 from humans in Czech, 4 from humans in China and 8 from animals in China) were used to trace the origins of foodborne *P. mirabilis* carrying *bla*_NDM_. The CSI Phylogeny 1.4 (https://cge.food.dtu.dk/services/CSIPhylogeny/ (accessed on 20 July 2022)) was used to obtain SNPs, and the phylogenetic tree was further visualized using iTOL v6 (https://itol.embl.de (accessed on 21 July 2022)).

### 2.7. Characterization of Plasmids Carrying bla_NDM_ or mcr

For the nanopore-sequenced *E. coli* M15061H and *P. mirabilis* M15061B, the complete plasmid sequences carrying *bla*_NDM_ were obtained, while the plasmid SPAdes tool (http://spades.bioinf.spbau.ru/plasmidSPAdes/ (accessed on 22 Jul 2022)) was used to extract contigs of plasmids carrying *bla*_NDM_ or *mcr* in the Illumina-sequenced isolates. For the sequenced transconjugants, contigs of plasmid were obtained after filtering the chromosomal DNA data of *E. coli* C600. The contigs of the reconstructed plasmid were aligned against the NCBI and the complete plasmids in this study to select the best match. The circular comparison of *bla*_NDM_ or *mcr-1*-positive plasmids was performed using the BRIG version 0.95 [39], and the plasmid linear alignment was analyzed with Easyfig version 2.1 [40].

### 2.8. Data Availability

The two *mcr-1*-bearing *E. coli* isolates carrying *bla*_NDM_ (M15071H, M15081H) and *bla*_NDM_-bearing *E. coli* M15061H have been deposited in BioProject PRJNA869497, which also contains the three *bla*_NDM_-positive transconjugants from *P. mirabilis*. The sequences of *bla*_NDM_-positive *P. mirabilis* M15061B and M15101B have been submitted to NCBI under the BioProject PRJNA869497.

## 3. Results

### 3.1. Virulence Genes and Concurrence of bla_NDM_-Positive P. mirabilis and E. coli from the Same Fresh Vegetables

In this study, carbapenem-resistant *P. mirabilis* isolates were found in five of the eight vegetable samples from one farmer’s market and one supermarket in Zhejiang Province. The five *P. mirabilis* isolates were from one tomato, two lettuce and two cucumber samples (Table 1). Notably, five carbapenem-resistant *E. coli* isolates were also isolated from the five fresh vegetables, respectively. All *P. mirabilis* isolates carried *bla*_NDM_, and *bla*_NDM-1_ was the most prevalent type (*n* = 4), while *bla*_NDM-5_ was found in all *E. coli* isolates (Table 1). Worryingly, except M15061H, the remaining four *E. coli* isolates co-harbored *mcr-1* and *bla*_NDM-5_. Notably, four vegetable samples carried both *bla*_NDM-1_ and *bla*_NDM-5_. All 15 virulence genes used for identifying diarrheagenic *E. coli* pathotypes were not found in our *E. coli* isolates, indicating that they were generic *E. coli.* Of the eight virulence genes detected, seven, including *hpmA*, *mrpA*, *ptA*, *ireA*, *zapA*, *pmfA* and *atfA*, were found in all the five *bla*_NDM_*-positive P. mirabilis* (Table 1).

### 3.2. Antimicrobial Resistance Patterns of bla_NDM_-Positive P. mirabilis and generic E. coli Isolates

The five *bla*_NDM_-positive *E. coli* isolates showed multidrug resistances, including resistance to β-lactams, aminoglycosides, tetracyclines and fluoroquinolones. Notably, the four *E. coli* isolates carrying both *mcr-1* and *bla*_NDM_ were also resistant to colistin (≥4 g/L) (Table 2). In the *bla*_NDM_-positive *P. mirabilis* isolates, M15061B was susceptible to fluoroquinolones, while the remaining isolates showed resistance to fluoroquinolones. All *P. mirabilis* isolates were also multidrug-resistant, including resistances to all β-lactams tested. In this study, only the *P. mirabilis* strain M15092B was resistant to amikacin. Luckily, all five *bla*_NDM_-positive *E. coli* strains remained susceptible to both amikacin and tigecycline.

### 3.3. MLST Typing and Transfer of bla_NDM_ or mcr-1

MLST analysis showed that all four *E. coli* isolates harboring both *mcr-1* and *bla*_NDM-5_ from three types of vegetables in two markets belonged to the ST6050 type, while isolate M15061H harboring only *bla*_NDM-5_ was the ST533 type (Table 1). Transconjugants containing *bla*_NDM_ were obtained in all 10 isolates in this study, and the carriage of *bla*_NDM_ resulted in all transconjugants being resistant to ampicillin, cefotaxime, meropenem and ceftazidime (Table 2). Only IncX3 replicon was found in the five *bla*_NDM_-positive transconjugants from *E. coli*, while the replicons in three *P. mirabilis*-derived transconjugants were untypable (Table 2). Notably, two plasmid replicons were found in transconjugants M15092BT. The *mcr-1* in the four ST6050 *E. coli* isolates could not be transferred into the recipient *E. coli* C600, although the conjugation experiment was performed three times.

### 3.4. Genomic Characteristics and Phylogenetic Analysis

WGS analysis showed that nanopore-sequenced *E. coli* M15061H possessed 16 types of resistance genes, including resistance to aminoglycosides (*aph(3′)-Ia*, *aph(6)-Id*, *aph(3″)-Ib* and *aadA5*), tetracyclines (*tet(A)*), sulfonamides (*sul2* and *sul1*), trimethoprim (*dfrA17*), peroxide (*sitABCD*), macrolides (*mph(A)*), β-lactams (*bla*_NDM-5_, *bla*_CTX-M-14_ and *bla*_TEM-1B_), fluoroquinolones (*gyrA*: (S83L, D87N), *parC*: S80I) and bleomycin (*ble*_MBL_) (Table 3). Both Illumina-sequenced *E. coli* isolates (M15071H and M15081H) from different types of vegetables had identical resistance genotypes, and both carried 21 resistance genes, including aminoglycosides (*aph(3′)-Ia*, *aph(6)-Id*, *aac(3)-IV*, *aph(3″)-Ib*, *aph(4)-Ia* and *aadA2*), tetracyclines (*tet(M)* and *tet(A)*), trimethoprim (*dfrA12*), fosfomycin (*fosA3*), amphenicol (*catB3*), rifamycin (*arr-3*), macrolides (*mph(A)*), fluoroquinolones (*gyrA*: (S83L, D87N), *parC*: S80I, and *aac(6′)-Ib-cr*), β-lactams (*bla*_CTX-M-3_, *bla*_NDM-5_ and *bla*_OXA-1_), bleomycin (*ble*_MBL_) and colistin (*mcr-1*) resistance genes (Table 3). The three sequenced *E. coli* isolates carried 14–21 virulence genes (Table 3).

The two sequenced *P. mirabilis* isolates M15061B and M15101B shared the same resistance genotypes, including aminoglycosides (*aph(4)-Ia*, *aac(3)-IV* and *aadA2*), tetracyclines (*tet(J)*), sulfonamides (*sul2* and *sul1*), amphenicol (*cat* and *floR*), rifamycin (*arr-3*), β-lactams (*bla*_NDM-1_), bleomycin (*ble*_MBL_) and lincosamide (*lnu(F)*) (Table 3). The three sequenced *bla*_NDM_-positive transconjugants derived from *P. mirabilis* shared some resistance genes, including sulfonamides (*sul2* and *sul1*), β-lactams (*bla*_NDM_) and bleomycin (*ble*_MBL_). All three transconjugants also harbored 3–7 diverse aminoglycosides resistance genes.

**Figure 1:**
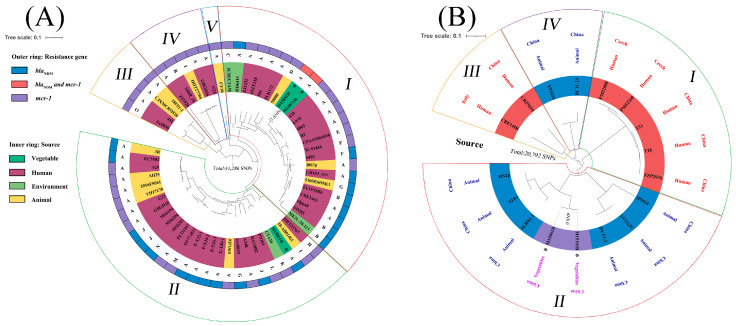
Phylogenetic tree of *E. coli* harboring *mcr-1* and/or *bla*_NDM_ (**A**) and *bla*_NDM_-positive *P. mirabilis* (**B**) by core genome sequences visualized using iTOL v6. (**A**) Phylogenetic tree of *E. coli* possessing *mcr-1* and/or *bla*_NDM_ from different origins and countries, including 57 genome sequences from NCBI and 3 isolates in this study. Strain M15061H in this study was used as a reference. A–O represent different countries, namely A: China, B: United States, C: Brazil, D: Lebanon, E: Denmark, F: Colombia, G: Switzerland, H: Italy, I: Germany, J: India, K: Ecuador, L: Thailand, M: Netherlands, N: United Kingdom and O: Bolivia. (**B**) Phylogenetic tree of *bla*_NDM_-positive *P. mirabilis* isolates from different origins and countries, including 15 genomes from NCBI and 2 isolates in this study. Strain M15061B in this study was used as a reference. Different clades were marked with different colored sectors. The genomes labeled with “*” were collected from vegetables in this study.

Based on the phylogenetic results of WGS, a total of 41,286 SNPs was obtained from these 60 *mcr-1* and/or *bla*_NDM_-positive *E. coli* isolates from different origins and counties. These 60 *E. coli* isolates were clustered into 5 clades (Figure 1A). The two *mcr-1*-positive NDM-producing ST6050 isolates M15071H and M15081H in this study belonged to clade I and had a limited number of variations (7 SNPs), although they were from different types of vegetables in the same market. The *bla*_NDM-5_-positive M15061H belonged to clade II and had a large number of variations from M15071H and M15081H (19,597 to 19,604 SNPs). Notably, both the *E. coli* isolates in our study, M15071H and M15081H, were closely clustered together with previously reported *bla*_NDM_/*mcr-1*-positive isolates (e.g., isolates A20, L935) from humans in different countries, especially China. The *bla*_NDM_/*mcr-1*-positive *E. coli* isolates from animals (isolates 50080, 1003p and 51008369SK1) and environments (isolates HD6415, ICBEC3AM and ME2L-20-113) from different countries, including China, were also clustered together with our two foodborne *E. coli* isolates (M15071H and M15081H) (Figure 1A). M15061H, carrying *bla*_NDM-5_ in this study, was also clustered together with previously reported *bla*_NDM_/*mcr-1*-bearing isolates from animals, humans and environments in different countries, including China.

A total of four phylogenetic clades were observed among the 17 NDM-producing *P. mirabilis* isolates, and 20,792 SNPs were obtained. Two *bla*_NDM_-positive *P. mirabilis* isolates (M15061B and M15101B) from different vegetable samples and different markets in this study were clustered together and had no SNP variation (0 SNPs) (Figure 1B). It is worth noting that the two vegetable-sourced *bla*_NDM_-positive *P. mirabilis* isolates in this study were clustered together with animal-sourced isolates from China, which were mainly located in clade II. All NDM-producing *P. mirabilis* isolates from humans were located in clades I and III, including clinical isolates from humans in China (Figure 1B).

### 3.5. Sequences of Plasmids Harboring mcr-1

**Figure 2:**
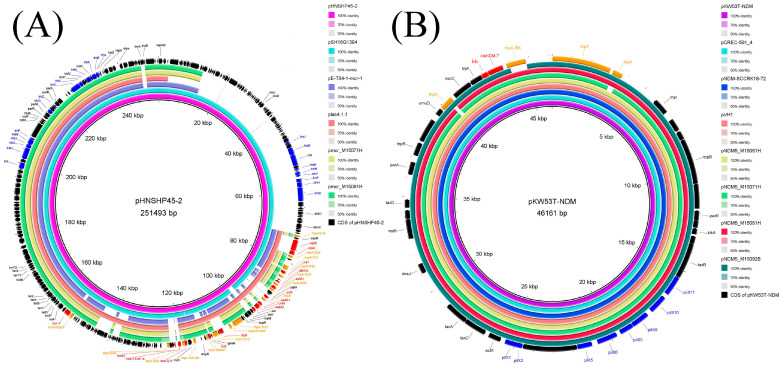
Circular alignment of IncHI2 *mcr-1*-bearing (**A**) or IncX3 *bla*_NDM-5_-bearing (**B**) plasmids by BRIG. (**A**) The plasmid pNSHNP45-2 (KU341381) (pink ring) from *E. coli* in pigs from China was set as a reference. The light blue, blue purple and orange rings represent pH16G1394 (NZ_MK477614) from clinical *Salmonella*, PE-T81-1-*mcr-1* (CP090269) from human *E. coli* and plas4.1.1 (NZ_CP047116) from *Salmonella* of chicken, respectively, in China. The yellow and green rings represent pmcr_M15071H and pmcr_M1508H in this study. (**B**) The plasmid pW53T-NDM (KX214669) (purple ring), from a urinary tract infection patient in Kuwait was set as a reference. The light blue, blue and orange rings represent pCREC-591_4 (CP024825) from ascites in a Korean patient, pNDM-SCCRK18-72 (MN565271) in *E. coli* in swine from China and pVH1 (CP028705) in vegetable-sourced *E. coli* from China, respectively. The yellow, green, red and dark green rings represent pNDM5_M15061H, pNDM5_M15071H, pNDM5_M15081H and pNDM5_M15092B in this study. The outer circle with black arrows represents annotation of the reference plasmids; among them, the red, blue and orange represent resistance genes, transfer-related genes and transposase genes, respectively.

The two Illumina-sequenced *E. coli* isolates carrying both *mcr-1* and *bla*_NDM_ (M15071H and M15081H) harbored IncHI2 plasmids with *mcr-1*. Both *mcr-1*-harboring IncHI2 plasmids pmcr_M15071H and pmcr_M15081H were about 177 kb in size and carried 13 types of resistance genes, which included colistin (*mcr-1*), β-lactams (*bla*_OXA-1_), trimethoprim (*dfrA12*), fluoroquinolones (two copies of *aac(6′)-Ib-cr*), aminoglycosides (*aph(4)-Ia*, *aph(3″)-Ib*, *aac(3)-IV*, *aph(6)-Id* and *aadA2*), tetracyclines (*tet(A*)), rifamycin (*arr-3*) and amphenicol (*catB3*) (Table 4). In both IncHI2 plasmids, except *mcr-1*, the remaining resistance genes were located in the MDR region, which contained multiple insertion sequences. One copy of IS*Apl1* was adjacent to *mcr-1* in the two *mcr-1*-harboring plasmids in the current study and a tellurium resistance region, including *terYXWZABCDEF,* was also detected in both plasmids (Figure 2A). Besides the MDR region, the two IncHI2 plasmids also possessed other backbone structures of the typical IncHI2-type plasmid pHNSHP45-2 (accession no. KU341381), including maintenance systems (*parA* and *parB*), plasmid replication (*repHI2* and *repHIA*) and transfer-associated regions (*trh* and *tra* series genes) (Figure 2A). The two *mcr-1*-positive plasmids of ~177 kb in our study were highly similar (≥98%) to *mcr-1*-positive plasmids pHNSHP45-2 (accession no. KU341381, 251 kb) and pSH16G1394 (accession no. NZ_MK477614, 252 kb) from pig-sourced *E. coli* and clinical *Salmonella* in China, respectively (Figure 2A). However, the plasmids pmcr_M15071H and pmcr_M15081H had only 70% of the sequences of typical *mcr-1*-harboring IncHI2 plasmids. When compared to pSH16G1394 and pHNSHP45-2, both plasmids (pmcr_M15071H and pmcr_M15081H) in our study lacked seven resistance genes, including *fosA3*, *bla*_CTX-M-14_, *floR*, *cmlA*, *sul3*, *oqxA* and *oqxB*. Notably, both the typical *mcr-1*-bearing IncHI2 plasmids (pSH16G1394 and pHNSHP45-2) contained transfer regions 1 (*traJ~trhG* genes) and transfer region 2 (*trhI~trhL* genes). However, transfer region 1 was not found in our *mcr-1*-bearing IncHI2 plasmids from vegetables (Figure 2A), which led to the failure of conjugation for our two plasmids. Both pmcr_M15071H and pmcr_M15081H were highly similar to pE-T84-1-mcr-1 (CP090269, 185 kb) (99.96% identity and 91% coverage) from clinical *E. coli* and plas4.1.1 (NZ_CP047116, 190 kb) (99.98% identity and 98% coverage) from chicken-sourced *Salmonella* in China (Figure 2A).

In order to investigate whether the backbone structures of *mcr-1*-bearing IncHI2 plasmids in unsequenced *E. coli* isolates (M15092H and M15101H) were similar to pmcr_M15071H, seven pairs of primers were designed. The seven pairs of primers were designed according to transfer region 2 (*trhE-trhK*, *trhV-trhC* and *traU-traN*), domain protein (DUF and VWA), heavy metal resistance (*terZ*-*terD*), tetracycline resistance (*tetR(A)*-*tet(A)*) and partial transfer region 1 (*traI*-*traG*) of pHNSHP45-2(KU341381) (Table 5 and Figure 2A). Except for transfer region 1 (*traI*-*traG*), the remaining six regions detected were all found in M15092H and M15101H, confirming that the backbone structure of the *mcr-1*-bearing plasmids in the two unsequenced *E. coli* as highly similar to pmcr_M15071H. Thus, all four IncHI2 plasmids carrying *mcr-1* in the current study were pmcr_M15071H-like plasmids and lacked transfer region 1.

### 3.6. Sequence Analysis of Plasmids Harboring bla_NDM_

The *bla*_NDM-5_-positive plasmid pNDM5_M15061H (CP102807, 46,161 bp) was IncX3 type. In addition to pNDM5_M15061H, isolate M15061H also carried seven additional plasmids, including one IncFII resistance plasmid (~152 kb) with 11 resistance genes (*aph(3′)-Ia*, *aph(3″)-Ib*, *aph(6)-Id*, *aadA5*, *bla*_TEM-1B_, *tet(A)*, *dfrA17*, *sul1*, *sul2*, *mph(A)* and *sitABCD*), and one IncY resistance plasmid (~152 kb) with *bla*_CTX-M-14_ (Table 4). In the four sequenced isolates or transconjugants with IncX3 replicon, *bla*_NDM_-carrying IncX3 plasmids were found in three *E. coli* isolates (pNDM5_M15061H, pNDM5_M15071H and pNDM5_M15081H) and one *P. mirabilis* (pNDM5_M15092B), and all these IncX3 plasmids were about 46 kb in size (Figure 2B and Table 4). All *bla*_NDM-5_-bearing IncX3 plasmids in this study carried two resistance genes (bleomycin resistance gene *ble*_MBL_ and *bla*_NDM-5_) (Table 4). Besides resistance genes, the four IncX3 plasmids also contained transfer-related region (*pilx* series of genes), maintenance system (*parB* and *parA*) and plasmid replication gene (*repB*) (Figure 2B). Notably, all four IncX3 plasmids in this study were similar (99.99% identity and 100% coverage) to the *bla*_NDM_-bearing IncX3 plasmids pKW53T (KX214669, 46,161 bp) from the urinary tract infection patient in Kuwait, pCREC-591_4 (CP024825, 46,161 bp) from patient ascites in Korea, pNDM-SCCRK18-72 (MN565271, 46,161 bp) from swine in China and our previously reported pVH1 (CP028705, 46,161 bp) from vegetables in China (Figure 2B).

**Figure 3:**
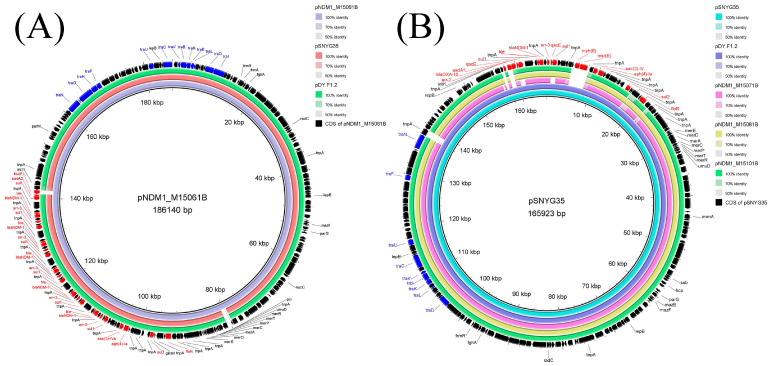
Circular sequence alignment of *bla*_NDM_-bearing untypable plasmids by BRIG. (**A**) The plasmid pNDM1_M15061B in this study (purple ring) was used as a reference. The light red and green rings represent pSNYG35 (CP047590) from *P. mirabilis* of chicken in China and pDY.F1.2 (CP046050) from *P. mirabilis* from swine in China. (**B**) The plasmid pSNYG35 (CP047590) (light blue ring) from *P. mirabilis* of chicken in China was used as a reference. The blue ring represents pDY.F1.2 (CP046050) from *P. mirabilis* from swine in China. The pink, orange and green rings represent pNDM1_M15071B, pNDM1_M15081B and pNDM1_M15101B in the current study, respectively. The black arrows in the outer circle represent annotation of the reference plasmid; among them, the red and blue represent resistance genes and transfer-related genes, respectively.

**Figure 4:**
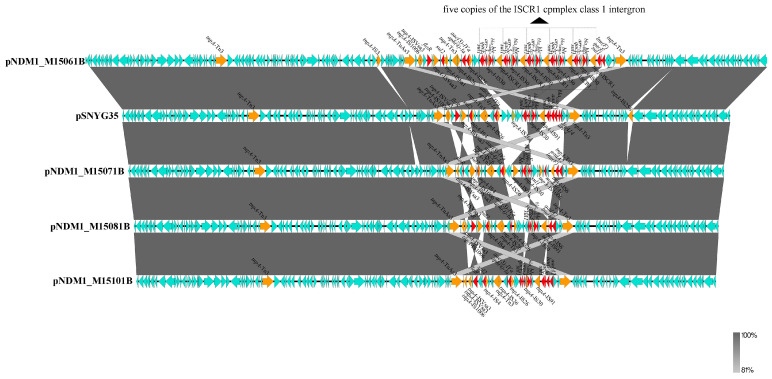
Linear alignment of *bla*_NDM-1_-bearing untypable plasmids from *P. mirabilis* isolates by Easyfig. Arrows indicate the directions of gene transcription. Regions with 100% nucleotide sequence homology are shown in grey.

In the nanopore-sequenced *P. mirabilis* isolate M15061B from lettuce, only one untypeable *bla*_NDM-1_-bearing plasmid pNDM1_M15061B (CP102813, 186,140 bp) was found. The pNDM1_M15061B carried ten types of resistance genes, including genes resistant to β-lactams (*bla*_NDM-1_), bleomycin (*ble*_MBL_), sulfonamides (*sul2* and *sul1*), aminoglycosides (*aadA2*, *aac(3)-IV* and *aph(4)-Ia*), rifamycin (*arr-3*), amphenicol (*floR*) and lincosamide (*lnu(F)*) (Table 4 and Figure 3A). The ten types of resistance genes were all located in the MDR region of ~61 kb on plasmid pNDM1_M15061B (Figure 3A and Figure 4). Resistance genes *ble*_MBL_, *bla*_NDM-1_, *arr-3* and sul1 were embedded in an IS*CR1* complex class 1 integron, and the structure of this class 1 integron (*sul1*-△*qacE*-*arr-3*-*bla*_NDM-1_-*ble*_MBL_-IS*CR1*) was about 6 kb in size (Figure 3A and Figure 4). Notably, there were five copies of this IS*CR1* complex class 1 integron on the plasmid pNDM1_M15061B in this study.

The plasmid contigs of the three untypable *bla*_NDM_-bearing plasmids pNDM_M15071B, pNDM_M15081B and pNDM_M15101B in *P. mirabilis* were also identified, and the sizes of the total plasmid contigs were about 158–163 kb. The two plasmids pNDM1_M15081B and pNDM1_M15101B also carried the same ten types of resistance genes as pNDM1_M15061B, while pNDM1_M15071B lacked *sul1* but had additional macrolides resistance gene *mph(A)* (Table 4). Notably, like pNDM1_M15061B, IS*CR1* complex class 1 integron (*sul1*-△*qacE*-*arr-3*-*bla*_NDM-1_-*ble*_MBL_-IS*CR1*) was also found on the three plasmids (pNDM_M15071B, pNDM_M15081B and pNDM_M15101B) (Figure 3B and Figure 4). All four untypable *bla*_NDM_-bearing plasmids from *P. mirabilis* isolates contained multiple transposases. Some resistance genes were flanked by two homologous IS sequences to form composite transposons, such as IS*Vsa3*-*floR*-IS*Vsa3* (~5 kb) and IS*26*-*aph(4)-Ia*-*acc(3)-IVa*-IS*26* (~4 kb) on these plasmids in this study, leading to the integration of resistance genes into chromosomes or plasmids (Figure 4). These four untypable *bla*_NDM_-bearing plasmids also carried a mercury resistance region containing *merRTPCADE*, transfer-related region (*tra* series genes) and maintenance systems such as *parB* (Figure 3A,B). All four untypable plasmids in *P. mirabilis* from fresh vegetables in the current study were highly similar (100% identity, >98% coverage) to plasmid pSNYG35 (CP047590, 165,923 bp) from *P. mirabilis* of chicken in China and pDY.F1.2 (CP046050, 165,918 bp) from *P. mirabilis* of swine in China. However, pNDM1_M15061B had four additional copies of IS*CR1* complex class 1 integron (*sul1*-*qacE*-*arr-3*-*bla*_NDM-1_-*ble*_MBL_-IS*CR1*, ~6 kb) compared with untypable *bla*_NDM_-bearing pSNYG35 (Figure 3 and Figure 4).

## 4. Discussion

It is well known that the *mcr*-carrying isolates or CRE pose a great threat to public health. To date, more than 40 subtypes of NDM have been reported in more than 60 species of bacteria from humans, animals and environments, with a high prevalence of NDM-producing Enterobacteriaceae, especially *E. coli* [3,41]. *P. mirabilis*, a member of Enterobacteriaceae, is an opportunistic pathogen for humans and animals. *P. mirabilis* is also a foodborne pathogen, and *P. mirabilis* from vegetables has been linked with foodborne illness [24]. However, there has been no report about *bla*_NDM_-positive *P. mirabilis* isolates from fresh vegetables, especially in China. Currently, studies about vegetable-sourced *E. coli* isolates co-carrying *mcr* and carbapenemases are still lacking. Here, we identified, for the first time, five *bla*_NDM_-positive *P. mirabilis* and five *bla*_NDM_-bearing *E. coli* concurrently from the same five fresh vegetables in China, and four of the five *E. coli* also carried *mcr-1*, confirming that fresh vegetables have been an important reservoir for *bla*_NDM_-positive Enterobacteriaceae, including *P. mirabilis*.

In this study, five fresh vegetable samples co-harbored *bla*_NDM_-positive *P. mirabilis* and *E. coli* carrying *bla*_NDM_, including two lettuces, two cucumbers and one tomato. The two cucumber samples from one market and one supermarket, respectively, harbored *P. mirabilis* with different subtypes of NDM. The two lettuces of different sampling origins also carried different ST types of *bla*_NDM_-positive *E. coli*. These results indicate that these five samples may be from different vegetable farms. In this study, ST6050 was the prevalent type of *E. coli* harboring both *mcr-1* and *bla*_NDM_ from fresh vegetables, different from that in our previous report [12], in which the two *E. coli* carrying both *mcr-1* and *bla*_NDM_ belonged to ST2847 and ST156, respectively. To our knowledge, this is the first report of ST6050 type of *E. coli* co-carrying *mcr-1* and *bla*_NDM_, especially in food. Carbapenemase-producing ST533 *E. coli* has appeared in patients [42]. Therefore, the ST533 *E. coli* carrying *bla*_NDM-5_ in lettuce will be a threat to human health. Luckily, all *bla*_NDM_-positive *E. coli* isolates in this study were not diarrheagenic *E. coli.* However, the *bla*_NDM_ and *mcr-1* within the generic *E. coli* isolate from vegetables eaten raw in the current study may be transferred to other foodborne pathogens or clinical pathogens.

Carbapenemases can confer high-level resistance to β-lactams, including carbapenems. For example, all 10 *bla*_NDM_-positive *E. coli* and *P. mirabilis* isolates in our study showed high-level resistance to meropenem (≥128 µg/mL). All five *bla*_NDM_-positive *E. coli* isolates from fresh vegetables in this study showed multidrug resistance. Luckily, all these five vegetable-sourced *bla*_NDM_-positive *E. coli* isolates were susceptible to tigecycline and amikacin, similar to our previously reported two *E. coli* isolates carrying both *bla*_NDM_ and *mcr-1* from vegetables [12]. These results suggest that amikacin and tigecycline may be good options for treating human infection caused by such bacteria. All *P. mirabilis* isolates in this study also showed multidrug resistances, but four isolates were susceptible to amikacin, suggesting that amikacin might be a good option for human infection caused by *bla*_NDM_-positive *P. mirabilis*. This finding was similar to that for clinical *P. mirabilis* carrying *bla*_NDM-1_ in China [21]. Clinical NDM-producing *P. mirabilis* has been reported in China [21], Tunisia [43], Portugal [44] and Austria [45]. The treatment of infections caused by *P. mirabilis* represents a particular challenge because of its intrinsic resistance to colistin and tetracyclines, including tigecycline. *P. mirabilis* is generally associated with food spoilage and can also cause foodborne illness [22,23]. In China, vegetables contaminated with *P. mirabilis* have been linked with foodborne illness [24]. All five *bla*_NDM_-positive *P. mirabilis* from fresh vegetables in this study possessed seven of the eight virulence genes, which were all often found in clinical *P. mirabilis* linked with urinary tract infection [30], indicating a potential threat to humans. Furthermore, *P. mirabilis* is notorious for its ability to actively disseminate antimicrobial resistance genes, including *bla*_NDM_ [21]. Thus, the concurrence of *bla*_NDM_-positive MDR *P. mirabilis* and *mcr-1*-positive *E. coli* producing NDM in fresh vegetables that are often eaten raw, poses a threat to human health.

IncX4 and IncI2 are the two major types of *mcr-1*-positive plasmids in *E. coli* from animals [46] and humans [47]. IncHI2 plasmids often carry multiple resistance genes [12], and IncHI2 plasmids possessing *mcr-1* have also been found in *E. coli* from cooked retail meat in China in recent years [48]. In our previous study, which only investigated *E. coli* carrying *mcr-1* among vegetables, IncX4 and IncI2 plasmids carrying *mcr-1* were also the two major plasmid types [49]. However, *mcr-1* was located on IncHI2 plasmids in all four vegetable-source *bla*_NDM_-positive *E. coli* isolates in this study. These data suggest that IncHI2-type plasmids play an important role in spreading *mcr-1* among vegetables in China, indicating further surveillance of IncHI2 plasmids carrying *mcr-1* is needed. Most previously reported *mcr-1*-harboring IncHI2 plasmids range from 210 to 260 kb in size and contain two transfer regions, as shown in [12], resulting in the transferability of these IncHI2 plasmids. However, the four *mcr-1*-positive IncHI2 plasmids in the current study were about 177 kb in size and did not harbor transfer region 1 (*traJ~trhG* genes), which might lead to the failure of conjugation for these plasmids. *mcr-1* has often been linked with one or two copies of IS*Apl1*, which plays an important role in spreading *mcr-1* [50]. One copy of IS*Apl1* was linked to *mcr-1* on the IncHI2 plasmids in our study, and Tn6330, an IS*Apl1*-flanked composite transposon was not found. Besides *mcr-1*, IncHI2 plasmids in the current study also carried additional twelve types of resistance genes, including β-lactams (*bla*_OXA-1_), trimethoprim (*dfrA12*), fluoroquinolones (*aac(6′)-Ib-cr*), aminoglycosides (*aph(4)-Ia*, *aph(3″)-Ib*, *aac(3)-IV*, *aph(6)-Id* and *aadA2*), tetracyclines (*tet(A*)), rifamycin (*arr-3*) and amphenicol (*catB3*). Most antimicrobials mentioned above are used for both humans and animals. Thus the adverse effects of these drugs on the spread of *mcr-1* should be paid more attention to.

IncX3 plasmids have been the main vector for the spread of *bla*_NDM_ among Enterobacteriaceae [51]. Notably, almost all IncX3 plasmids carrying *bla*_NDM_ carried the *bla*_NDM-5_ gene, while the *E. coli* isolates carrying other subtypes of *bla*_NDM_ from animals harbored other replicon types rather than IncX3 in a previous study [3], consistent with the findings in our study that all IncX3 plasmids in the six isolates (*E. coli* and *P. mirabilis*) from fresh vegetables carried *bla*_NDM-5_ and *P. mirabilis* isolates with *bla*_NDM-1_ harbored no IncX3 replicon. The environment surrounding *bla*_NDM-5_ contained the mobile element IS*5*, suggesting that *bla*_NDM-5_ was recombined into IncX3 plasmids by insertion or transposition. A retrospective analysis of IncX3 plasmids in China showed that the backbone of IncX3 plasmids has been highly conserved [52]. The *bla*_NDM-5_-positive IncX3 plasmids in this study were transferable and also had a highly similar backbone structure, although these plasmids were from bacteria of different genera, different ST types or different types of vegetables. These results suggest that the horizontal transfer of similar IncX3 plasmids might be responsible for the spread of *bla*_NDM-5_ among vegetables in China. All IncX3 plasmids from fresh vegetables in our study were similar to the *bla*_NDM_-positive IncX3 plasmids pKW53T (KX214669, 46,161 bp) from the urinary tract infection patient in Kuwait, pCREC-591_4 (CP024825, 46,161 bp) from patient ascites in Korea and pNDM-SCCRK18-72 (MN565271, 46,161 bp) from swine in China. These results suggest that the transferable *bla*_NDM_-bearing IncX3 plasmids in fresh vegetables might be derived from animals and then transmitted to humans through the food chain.

Unlike *E. coli* and *Klebsiella pneumoniae*, in which IncX3 and IncFII plasmids were the two main vectors for spreading *bla*_NDM_, the *bla*_NDM-1_-bearing plasmids in *P. mirabilis* isolates were often assigned to an unknown incompatibility group [53]. In China, *bla*_NDM-1_ was also located on plasmids with unknown replicon type in *P. mirabilis* isolates from chicken [54]. Similarly, we obtained four untypable plasmids carrying *bla*_NDM-1_ from *P. mirabilis* isolates in vegetables, further confirming the association of untypable plasmids and *bla*_NDM-1_ in *P. mirabilis* isolates. The four untypable *bla*_NDM-1_-bearing plasmids in *P. mirabilis* isolates from different types of vegetables or markets in our study were highly similar to untypable *bla*_NDM-1_-positive pSNYG35 from cloacal swabs of broilers in China, further confirming the previous finding that the family of *bla*_NDM-1_-carrying untypable plasmids in *P. mirabilis* shares high homologous backbones [53]. This result indicates that *bla*_NDM-1_-positive plasmids in *P. mirabilis* from vegetables may come from isolates from animals. Insertion sequence common region (IS*CR*) is an IS*91*-like element that could mobilize adjacent sequences through the mechanism “rolling-circle replication” [55], and IS*CR1* is a well-established gene capture system. *bla*_NDM−1_ could be disseminated by a circular IS*CR1*-*bla*_NDM−1_ element [56], and in this study, the untypable plasmids in *P. mirabilis* contained IS*CR1* complex class 1 integron (*sul1*-△*qacE*-*arr-3*-*bla*_NDM-1_-*ble*_MBL_-IS*CR1*). The IS*CR1* complex class 1 integron containing *sul1*-△*qacE*-*arr-3*-*bla*_NDM-1_-*ble*_MBL_-IS*CR1/*IS*91* was also found on plasmid pSNYG35 in *P. mirabilis* isolated from cloacal swabs of broilers in China [53]. These results deepen our conjecture that the plasmids or *bla*_NDM-1_ in *P. mirabilis* from vegetables may have come from animals via plasmid transfer or gene capture. Notably, the nanopore-sequenced data confirmed that plasmid pNDM1_M15061B in *P. mirabilis* contained five copies of the IS*CR1* complex class 1 integron (*sul1*-△*qacE*-*arr-3*-*bla*_NDM-1_-*ble*_MBL_-IS*CR1*) in this study. We speculate that the IS*CR1* element captures multiple antimicrobial resistance genes, including *sul1*-△*qacE*-*arr-3*-*bla*_NDM-1_-*ble*_MBL_, by several steps, resulting in the formation of pNDM1_M15061B. The meropenem MICs of previously reported *bla*_NDM-1_-positive *P. mirabilis* from animals ranged from 32 to 64 µg/mL [54], and those from humans were from 2 to 64 µg/mL [21]. *P. mirabilis* (64 µg/mL) from humans was confirmed to possess two copies of *bla*_NDM-1_. The meropenem MICs of M15061 and its transconjugant M15061BT in this study were ≥128 µg/mL, which might be attributed to the fact that both M15061 and its transconjugant M15061BT carried five copies of IS*CR1* complex class 1 integron-containing *bla*_NDM-1_. The meropenem MICs of the remaining four *P. mirabilis* and their transconjugants in our study were also ≥128 µg/mL, indicating that the remaining four *P. mirabilis* may also harbor at least one copy of IS*CR1* complex class 1 integron-containing *bla*_NDM-1_, although the structure of multiple copies of IS*CR1* complex class 1 integron in these isolates were not obtained only from Illumina-sequenced data.

The sequenced *mcr-1*-positive NDM-producing ST6050 isolates (M15071H and M15081H) in this study were highly similar (7 SNPs), although they were isolated from different types of vegetables in the same market, indicating a very close genetic relationship between these two isolates. Notably, the NDM-producing *E. coli* isolates from fresh vegetables were clustered together with previously reported *bla*_NDM_/*mcr-1*-positive isolates from animals, humans and environments in different countries, including China. These results suggest that the NDM-producing *E. coli* isolates with *mcr-1* from fresh vegetables in our study may be derived from animals through fecal fertilization and transferred to humans. The *bla*_NDM_-positive *P. mirabilis* isolates obtained from vegetables in this study had no SNP variation and were also clustered with animal-sourced isolates from China. These results suggest that *bla*_NDM_-positive *P. mirabilis* isolates in vegetables may be derived from animals because a relatively high prevalence of *P. mirabilis* isolates carrying *bla*_NDM-1_ has already been found in chickens from China [54].

## 5. Conclusions

In conclusion, we reported, for the first time, five *bla*_NDM_-positive *P. mirabilis* and five *bla*_NDM_-bearing generic *E. coli* concurrently from the same five fresh vegetables in China, and four of the five *E. coli* also carried *mcr-1. bla*_NDM-5_ in all *E. coli* isolates, and *P. mirabilis* carrying *bla*_NDM-5_ from fresh vegetables was located on similar IncX3 transferable plasmids, while similarly untypable transferable plasmids were the carriers of *bla*_NDM-1_ in *P. mirabilis* isolates from different types of vegetables or markets. *mcr-1* in all *bla*_NDM_-_5_-positive *E. coli* was located on similarly non-conjugative IncHI2 MDR plasmids lacking a transfer region. Notably, IS*CR1* complex class 1 integron capable of capturing *bla*_NDM-1_ was found on transferable untypable plasmids from *P. mirabilis* in this study, and five copies of IS*CR1* complex class 1 integron were even found in one *P. mirabilis*. Plasmid comparison and phylogenetic analysis revealed that the *bla*_NDM_-positive *P. mirabilis* and *bla*_NDM_-positive *E. coli* in fresh vegetables might be derived from animals by fecal fertilization and could be transmitted to humans through the food chain. Fresh retail vegetables might have been underestimated vehicles of *E. coli* and *P. mirabilis* in spreading resistance genes, including both *bla*_NDM_ and *mcr-1*. The concurrence of *bla*_NDM_-positive *P. mirabilis* and *E. coli* possessing both *mcr-1* and *bla*_NDM_ in different types of fresh vegetables eaten raw is alarming and threatens food safety. Sustained surveillance of resistance in foodborne pathogens in the food chain, especially fresh vegetables, is urgent for preventing the transmission of MCR-producing and/or NDM-producing Enterobacteriaceae to ensure the health of food consumers.

## Figures and Tables

**Table 1:** Origins, resistance genes and virulence genes of five *P. mirabilis* carrying *bla*_NDM_ and five *bla*_NDM_-positive *E. coli* from the same five fresh vegetables.

Sources	Samples	*E. coli*				*P. mirabilis*		
		Strains	Resistance Genes	STs	Diarrheagenic Virulence Genes	Strains	Resistance Genes	Virulence Genes
Market	lettuce	M15061H	*bla*_NDM-5_	ST553	Not found	M15061B	*bla*_NDM-1_	*hpmA, mrpA, ptA, ireA, zapA, pmfA*, *atfA*
	tomato	M15071H	*mcr-1, bla*_NDM-5_	ST6050	Not found	M15071B	*bla*_NDM-1_	*hpmA, mrpA, ptA, ireA, zapA, pmfA*, *atfA*
	cucumber	M15081H	*mcr-1, bla*_NDM-5_	ST6050	Not found	M15081B	*bla*_NDM-1_	*hpmA, mrpA, ptA, ireA, zapA, pmfA*, *atfA*
Supermarket	cucumber 1	M15092H	*mcr-1, bla*_NDM-5_	ST6050	Not found	M15092B	*bla*_NDM-5_	*hpmA, mrpA, ptA, ireA, zapA, pmfA*, *atfA*
	lettuce	M15101H	*mcr-1, bla*_NDM-5_	ST6050	Not found	M15101B	*bla*_NDM-1_	*hpmA, mrpA, ptA, ireA, zapA, pmfA*, *atfA*
	green pepper	-				-		
	cucumber 2	-				-		
	tomato	-				-		

**Table 2:** Resistance characteristics of the 10 *bla*_NDM_-positive isolates and their transconjugants harboring *bla*_NDM_ in this study.

Strains	Replicons in Transconjugants	Resistance Genes	MICs (μg/mL)						Other Resistance Profiles
			COL	TIG	MEM	CTX	CAZ	AMP	
M15061H	-	*bla*_NDM-5_	0.5	0.25	>128	>256	>256	>256	STR, KAN, TET, NAL, CIP
M15071H	-	*mcr-1, bla*_NDM-5_	4	0.25	>128	>256	>256	>256	STR, KAN, TET, LEV, NAL, CIP
M15081H	-	*mcr-1, bla*_NDM-5_	4	0.5	>128	>256	>256	>256	STR, KAN, TET, LEV, NAL, CIP
M15092H	-	*mcr-1, bla*_NDM-5_	4	0.125	>128	>256	>256	>256	STR, KAN, TET, LEV, NAL, CIP
M15101H	-	*mcr-1, bla*_NDM-5_	4	0.25	>128	>256	>256	>256	STR, KAN, TET, LEV, NAL, CIP
M15061B	-	*bla*_NDM-1_	NA	NA	>128	128	>256	>256	STR, KAN, TET
M15071B	-	*bla*_NDM-1_	NA	NA	>128	>256	>256	>256	STR, KAN, TET, NAL, CIP
M15081B	-	*bla*_NDM-1_	NA	NA	>128	>256	>256	>256	STR, KAN, TET, LEV, NAL, CIP
M15092B	-	*bla*_NDM-5_	NA	NA	>128	>256	>256	>256	STR, KAN, AMK, TET, NAL, CIP
M15101B	-	*bla*_NDM-1_	NA	NA	>128	256	>256	>256	STR, KAN, TET, NAL, CIP
M15061HT	X3	*bla*_NDM-5_	<0.125	0.125	>128	128	256	>256	STR
M15071HT	X3	*bla*_NDM-5_	<0.125	0.25	>128	256	128	>256	STR
M15081HT	X3	*bla*_NDM-5_	<0.125	0.125	>128	256	256	>256	STR
M15092HT	X3	*bla*_NDM-5_	<0.125	0.125	>128	256	256	>256	STR
M15101HT	X3	*bla*_NDM-5_	<0.125	0.25	>128	256	256	>256	STR
M15061BT	UT	*bla*_NDM-1_	<0.125	0.125	128	64	256	>256	STR
M15071BT	F24:A-:B6	*bla*_NDM-1_	<0.125	0.125	128	64	256	>256	STR
M15081BT	UT	*bla*_NDM-1_	<0.125	0.25	128	128	256	>256	STR
M15092BT	X3, F24:A-:B6	*bla*_NDM-5_	<0.125	0.5	>128	128	256	>256	STR, TET
M15101BT	UT	*bla*_NDM-1_	<0.125	0.125	>128	256	256	>256	STR
C600	-	*-*	<0.125	0.125	0.125	0.031	0.062	4	STR

COL, colistin; TIG, tigecycline; AMP, ampicillin; MEM, meropenem; CTX, cefotaxime; CAZ, ceftazidime; NAL, nalidixic acid; CIP, ciprofloxacin; STR, streptomycin; KAN, kanamycin; AMK, amikacin; LEV, levofloxacin; TET, tetracycline; UT, untypable; NA, not applicable because of intrinsic resistance.

**Table 3:** Antimicrobial resistance genes of obtained isolates from WGS sequencing.

Strains	Origins	Strategies of Sequencing	Resistance Genes	Virulence Genes
*E. coli*				
M15061H	lettuce	MinION + HiSeq	*aph(3′)-Ia*, *aph(6)-Id*, *aph(3″)-Ib*, *aadA5*, *tet(A)*, *sul2*, *sul1*, *dfrA17*, *sitABCD*, *mph(A)*, *bla*_NDM-5_, *bla*_CTX-M-14_, *bla*_TEM-1B_, *ble*_MBL_, *gyrA:* (S83L,D87N), *parC:* S80I	*cma*, *csgA*, *cvaC*, *fdeC*, *fimH*, *gad*, *hlyE*, *hlyF*, *iroN*, *iss*, *lpfA*, *nlpI*, *ompT*, *sitA*, *terC*, *traJ*, *traT*, *yehA*, *yehB*, *yehC*, *yehD*
M15071H	tomato	HiSeq	*aph(3′)-Ia*, *aph(6)-Id*, *aac(3)-IV*, *aph(3″)-Ib*, *aph(4)-Ia*, *aac(6′)-Ib-cr*, *aadA2*, *tet(M*), *tet(A)*, *dfrA12*, *fosA3*, *catB3*, *arr-3*, *mph(A)*, *bla*_NDM-5_, *bla*_CTX-M-3_, *bla*_OXA-1_, *ble*_MBL_, *mcr-1*, *gyrA:* (S83L,D87N)*, parC:* S80I	*astA*, *csgA*, *fimH*, *gad*, *hlyE*, *nlpI*, *ompT*, *terC*, *traJ*, *traT*, *yehA*, *yehB*, *yehC*, *yehD*
M15081H	cucumber	HiSeq	*aph(3′)-Ia*, *aph(6)-Id*, *aac(3)-IV*, *aph(3″)-Ib*, *aph(4)-Ia*, *aac(6′)-Ib-cr*, *aadA2*, *tet(M*), *tet(A*), *dfrA12*, *fosA3*, *catB3*, *arr-3*, *mph(A)*, *bla*_NDM-5_, *bla*_CTX-M-3_, *bla*_OXA-1_, *ble*_MBL_, *mcr-1*, *gyrA:* (S83L,D87N)*, parC:* S80I	*astA*, *csgA*, *fimH*, *gad*, *hlyE*, *nlpI*, *ompT*, *terC*, *traJ*, *traT*, *yehA*, *yehB*, *yehC*, *yehD*
*P. mirabilis*				
M15061B	lettuce	MinION + HiSeq	*aph(4)-Ia*, *aac(3)-IV*, *aadA2*, *tet(J*), *sul2*, *sul1*, *cat, floR*, *arr-3*, *bla*_NDM-1_, *ble*_MBL_, *lnu(F)*	*-*
M15101B	lettuce	HiSeq	*aph(4)-Ia*, *aac(3)-IV*, *aadA2*, *tet(J)*, *sul2*, *sul1*, *cat*, *floR*, *arr-3*, *bla*_NDM-1_, *ble*_MBL_, *lnu(F)*	*-*
transconjugants				
M15071BT	-	HiSeq	*aph(6)-Id*, *aph(3′)-Ia*, *aph(3″)-Ib*, *aac(3)-IV*, *aph(4)-Ia*, *aadA2*, *aadA5*, *tet(A*), *sul2*, *sul1*, *dfrA17*, *sitABCD*, *floR*, *arr-3*, *mph(A)*, *bla*_NDM-1_, *bla*_TEM-1B_, *ble*_MBL_, *lnu(F)*	-
M15081BT	-	HiSeq	*aac(3)-IV*, *aph(4)-Ia*, *aadA2*, *sul2*, *sul1*, *floR*, *arr-3, bla*_NDM-1_, *ble*_MBL_, *lnu(F)*	-
M15092BT	-	HiSeq	*aph(6)-Id*, *aph(3′)-Ia*, *aph(3″)-Ib*, *aadA5*, *tet(A*), *sul2*, *sul1*, *dfrA17*, *sitABCD*, *mph(A), bla*_NDM-5_, *bla*_TEM-1B_, *ble*_MBL_	-

**Table 4:** Characteristics of plasmids harboring *mcr-1* or *bla*_NDM_ from fresh vegetables.

Strains	Plasmids	Resistance Genes	Plasmids Carrying *mcr-1* or *bla*_NDM_	
			Replicon Type	Size (kb)
M15061H	pNDM5_M15061H	*bla*_NDM-5_, *ble*_MBL_	X3	~46
	pTEM-1B_M15061H	*aph(3′)-Ia, aph(3″)-Ib*, *aph(6)-Id*, *aadA5*, *bla*_TEM-1B_, *tet(A)*, *dfrA17*, *sul1*, *sul2*, *mph(A), sitABCD*	FII	~152
	pCTX-M-14_M15061H	*bla*_CTX-M-14_	Y	~152
	p1_M15061H		I1-I	~90
	p2_M15061H		UT	~5
	p3_M15061H		UT	~3
	p4_M15061H		UT	~3
	p5_M15061H		UT	~2
M15071H	pmcr_M15071H	*mcr-1*, *bla_OXA-1_*, *dfrA12*, *aac(6′)-Ib-cr*, *aph(3′)-Ia*, *aph(4)-Ia*, *aac(3)-IV*, *aph(3″)-Ib*, *aph(6)-Id*, *aadA2*, *tet(A)*, *arr-3*, *catB3*	HI2	~177
	pNDM5_M15071H	*bla*_NDM-5_, *ble*_MBL_	X3	~46
M15081H	pmcr_M15081H	*mcr-1*, *bla_OXA-1_*, *dfrA12*, *aac(6′)-Ib-cr*, *aph(3′)-Ia*, *aph(4)-Ia*, *aac(3)-IV*, *aph(3″)-Ib*, *aph(6)-Id*, *aadA2*, *tet(A)*, *arr-3*, *catB3*	HI2	~177
	pNDM5_M15081H	*bla*_NDM-5_, *ble*_MBL_	X3	~46
M15092H	pmcr_M15092H	*mcr-1*, *bla_OXA-1_*, *dfrA12*, *aac(6′)-Ib-cr*, *aph(3′)-Ia*, *aph(4)-Ia*, *aac(3)-IV*, *aph(3″)-Ib*, *aph(6)-Id*, *aadA2*, *tet(A)*, *arr-3*, *catB3*	HI2	~177
	pNDM5_M15092H	*bla*_NDM-5_, *ble*_MBL_	X3	~46
M15101H	pmcr_M15101H	*mcr-1*, *bla_OXA-1_*, *dfrA12*, *aac(6′)-Ib-cr*, *aph(3′)-Ia*, *aph(4)-Ia*, *aac(3)-IV*, *aph(3″)-Ib*, *aph(6)-Id*, *aadA2*, *tet(A)*, *arr-3*, *catB3*	HI2	~177
	pNDM5_M15101H	*bla*_NDM-5_, *ble*_MBL_	X3	~46
M15061B	pNDM1_M15061B	*bla*_NDM-1_, *ble*_MBL_, *sul1*, *sul2*, *aadA2*, *aac(3)-IV*, *aph(4)-Ia*, *arr-3*, *folR, lnu(F)*	UT	~186
M15071B	pNDM1_M15071B	*bla*_NDM-1_, *ble*_MBL_, *sul2*, *aadA2*, *aac(3)-IV*, *aph(4)-Ia*, *mph(A)*, *arr-3*, *floR*, *lnu(F)*	UT	~163
M15081B	pNDM1_M15081B	*bla*_NDM-1_, *ble*_MBL_, *sul1*, *sul2*, *aadA2*, *aac(3)-IV*, *aph(4)-Ia*, *arr-3*, *folR, lnu(F)*	UT	~160
M15092B	pNDM5_M15092B	*bla*_NDM-5_, *ble*_MBL_	X3	~46
M15101B	pNDM1_M15101B	*bla*_NDM-1_, *ble*_MBL_, *sul1*, *sul2*, *aadA2*, *aac(3)-IV*, *aph(4)-Ia*, *arr-3*, *folR, lnu(F)*	UT	~158

**Table 5:** Primer sequences used for the detection of pmcr_M15071H-like plasmids.

Primers	DNA Sequence (5′→3′)	Target Genes	Products Size (bp)
trhE-trhK-F	AACGGTGATCTTGAACAGTC	*trhE* and *trhK*	1000
trhE-trhK-R	ACGGTAGGGAGATCAGTTG		
trhV-trhC-F	CAACAGGGGAAAGTAATGAG	*trhV* and *trhC*	999
trhV-trhC-R	GTTTGAAGTAACGATGCTCAG		
traU-traN-F	CAACACTAATCAGCCAATGAC	*traU* and *traN*	992
traU-traN-R	GATTAAGATTAGCGGATTCGG		
DUF-VWA-F	GATTGAACGAGAGTTTCAGG	*DUF* and *VWA*	980
DUF-VWA-R	ACAGGATCAAAATACGGTCC		
terZ-terD-F	GAGTTAACCAGTCGACGC	*terZ* and *terD*	997
terZ-terD-R	TAAACGCCAGGTATTCAACG		
tetR(A)-tet(A)-F	TTCTATCTGCGATTGGACCC	*tetR(A)* and *tet(A)*	872
tetR(A)-tet(A)-R	CTAGTATGACGTCTGTCGC		
traG-traI-F	AAGCTTATCGACCTCTTTCG	*traG* and *traI*	993
traG-traI-R	AATGCAAAGCATACAGCATC		

## Data Availability

Data are contained within the article or supplementary material.

## Supplementary Materials

The following supporting information can be downloaded at: https://www.mdpi.com/article/10.3390/foods12030492/s1, Table S1: Information about strains used for *Escherichia coli* evolutionary tree from NCBI database.

## References

[1] van Duin D., Doi Y. (2017). The global epidemiology of carbapenemase-producing Enterobacteriaceae. Virulence.

[2] Zhang R., Liu L., Zhou H., Chan E.W., Li J., Fang Y., Li Y., Liao K., Chen S. (2017). Nationwide Surveillance of Clinical Carbapenem-resistant Enterobacteriaceae (CRE) Strains in China. EBioMedicine.

[3] Zhai R., Fu B., Shi X., Sun C., Liu Z., Wang S., Shen Z., Walsh T.R., Cai C., Wang Y. (2020). Contaminated in-house environment contributes to the persistence and transmission of NDM-producing bacteria in a Chinese poultry farm. Environ. Int..

[4] CDC (2019). Antibiotic Resistance Threats in the United States, 2019.

[5] Delgado-Blas J.F., Agui C.V., Rodriguez E.M., Serna C., Montero N., Saba C.K.S., Gonzalez-Zorn B. (2022). Dissemination Routes of Carbapenem and Pan-Aminoglycoside Resistance Mechanisms in Hospital and Urban Wastewater Canalizations of Ghana. mSystems.

[6] Li F., Cheng P., Li X., Liu R., Liu H., Zhang X. (2022). Molecular Epidemiology and Colistin-Resistant Mechanism of *mcr*-Positive and *mcr*-Negative *Escherichia coli* Isolated From Animal in Sichuan Province, China. Front. Microbiol..

[7] Wang C., Feng Y., Liu L., Wei L., Kang M., Zong Z. (2020). Identification of novel mobile colistin resistance gene *mcr-10*. Emerg. Microbes Infect..

[8] Xu T., Xue C.X., Chen Y., Huang J., Wu W., Lu Y., Huang Q., Chen D., Zhou K. (2022). Frequent convergence of *mcr-9* and carbapenemase genes in *Enterobacter cloacae* complex driven by epidemic plasmids and host incompatibility. Emerg. Microbes Infect..

[9] Aklilu E., Harun A., Singh K.K.B. (2022). Molecular characterization of *bla*_NDM_, *bla*_OXA-48_, *mcr-1* and *bla*_TEM-52_ positive and concurrently carbapenem and colistin resistant and extended spectrum beta-lactamase producing *Escherichia coli* in chicken in Malaysia. BMC Vet. Res..

[10] Liu X., Li R., Dong N., Ye L., Chan E.W., Chen S. (2021). Complete Genetic Analysis of Plasmids Carried by Two Nonclonal *bla*_NDM-5_- and *mcr-1*-Bearing *Escherichia coli* Strains: Insight into Plasmid Transmission among Foodborne Bacteria. Microbiol. Spectr..

[11] Jung Y., Jang H., Matthews K.R. (2014). Effect of the food production chain from farm practices to vegetable processing on outbreak incidence. Microb. Biotechnol..

[12] Liu B.T., Song F.J. (2019). Emergence of two *Escherichia coli* strains co-harboring *mcr-1* and *bla*_NDM_ in fresh vegetables from China. Infect. Drug Resist..

[13] van Hoek A.H., Veenman C., van Overbeek W.M., Lynch G., de Roda Husman A.M., Blaak H. (2015). Prevalence and characterization of ESBL- and AmpC-producing Enterobacteriaceae on retail vegetables. Int. J. Food Microbiol..

[14] Chelaghma W., Loucif L., Bendjama E., Cherak Z., Bendahou M., Rolain J.M. (2022). Occurrence of Extended Spectrum Cephalosporin-, Carbapenem- and Colistin-Resistant Gram-Negative Bacteria in Fresh Vegetables, an Increasing Human Health Concern in Algeria. Antibiotics.

[15] Jones-Dias D., Manageiro V., Ferreira E., Barreiro P., Vieira L., Moura I.B., Canica M. (2016). Architecture of Class 1, 2, and 3 Integrons from Gram Negative Bacteria Recovered among Fruits and Vegetables. Front. Microbiol..

[16] Zurfuh K., Poirel L., Nordmann P., Nuesch-Inderbinen M., Hachler H., Stephan R. (2016). Occurrence of the plasmid-borne *mcr-1* colistin resistance gene in ESBL-producing Enterobacteriacae in river water and imported vegetable samples in Switzerland. Antimicrob. Agents Chemother..

[17] Oh S.S., Song J., Kim J., Shin J. (2020). Increasing prevalence of multidrug-resistant *mcr-1*-positive *Escherichia coli* isolates from fresh vegetables and healthy food animals in South Korea. Int. J. Infect. Dis..

[18] Luo J., Yao X., Lv L., Doi Y., Huang X., Huang S., Liu J.H. (2017). Emergence of *mcr-1* in *Raoultella ornithinolytica* and *Escherichia coli* Isolates from Retail Vegetables in China. Antimicrob. Agents Chemother..

[19] Soliman A.M., Nariya H., Tanaka D., Yu L., Hisatsune J., Kayama S., Kondo K., Sugai M., Shimamoto T., Shimamoto T. (2021). Vegetable-Derived Carbapenemase-Producing High-Risk *Klebsiella pneumoniae* ST15 and *Acinetobacter baumannii* ST2 Clones in Japan: Coexistence of *bla*_NDM-1_, *bla*_OXA-66_, *bla*_OXA-72_, and an AbaR4-Like Resistance Island in the Same Sample. Appl. Environ. Microbiol..

[20] Liu B.T., Zhang X.Y., Wan S.W., Hao J.J., Jiang R.D., Song F.J. (2018). Characteristics of Carbapenem-Resistant Enterobacteriaceae in Ready-to-Eat Vegetables in China. Front. Microbiol..

[21] Yang L., He H., Chen Q., Wang K., Lin Y., Li P., Li J., Liu X., Jia L., Song H. (2022). Nosocomial Outbreak of Carbapenemase-Producing *Proteus mirabilis* With Two Novel *Salmonella* Genomic Island 1 Variants Carrying Different *bla*_NDM-1_ Gene Copies in China. Front. Microbiol..

[22] Keisam S., Tuikhar N., Ahmed G., Jeyaram K. (2019). Toxigenic and pathogenic potential of enteric bacterial pathogens prevalent in the traditional fermented foods marketed in the Northeast region of India. Int. J. Food Microbiol..

[23] Nyenje M.E., Odjadjare C.E., Tanih N.F., Green E., Ndip R.N. (2012). Foodborne pathogens recovered from ready-to-eat foods from roadside cafeterias and retail outlets in Alice, Eastern Cape Province, South Africa: Public health implications. Int. J. Environ. Res. Public Health.

[24] Gong Z., Shi X., Bai F., He X., Zhang H., Li Y., Wan Y., Lin Y., Qiu Y., Chen Q. (2019). Characterization of a Novel Diarrheagenic Strain of *Proteus mirabilis* Associated With Food Poisoning in China. Front. Microbiol..

[25] Mollet C., Drancourt M., Raoult D. (1997). *rpoB* sequence analysis as a novel basis for bacterial identification. Mol. Microbiol..

[26] Poirel L., Walsh T.R., Cuvillier V., Nordmann P. (2011). Multiplex PCR for detection of acquired carbapenemase genes. Diagn. Microbiol. Infect. Dis..

[27] Borowiak M., Baumann B., Fischer J., Thomas K., Deneke C., Hammerl J.A., Szabo I., Malorny B. (2020). Development of a Novel *mcr-6* to *mcr-9* Multiplex PCR and Assessment of *mcr-1* to *mcr-9* Occurrence in Colistin-Resistant *Salmonella enterica* Isolates From Environment, Feed, Animals and Food (2011–2018) in Germany. Front. Microbiol..

[28] Antikainen J., Tarkka E., Haukka K., Siitonen A., Vaara M., Kirveskari J. (2009). New 16-plex PCR method for rapid detection of diarrheagenic *Escherichia coli* directly from stool samples. Eur. J. Clin. Microbiol. Infect. Dis..

[29] Meraz I.M., Jiang Z.D., Ericsson C.D., Bourgeois A.L., Steffen R., Taylor D.N., Hernandez N., DuPont H.L. (2008). Enterotoxigenic *Escherichia coli* and diffusely adherent *E. coli* as likely causes of a proportion of pathogen-negative travelers’ diarrhea—A PCR-based study. J. Travel Med..

[30] de Oliveira W.D., Barboza M.G.L., Faustino G., Inagaki W.T.Y., Sanches M.S., Kobayashi R.K.T., Vespero E.C., Rocha S.P.D. (2021). Virulence, resistance and clonality of *Proteus mirabilis* isolated from patients with community-acquired urinary tract infection (CA-UTI) in Brazil. Microb. Pathog..

[31] (2019). Performance Standards for Antimicrobial Susceptibility Testing.

[32] EUCAST (2019). Breakpoint Tables for Interpretation of MICs and Zone Diameters, Version 9.0.

[33] Wirth T., Falush D., Lan R., Colles F., Mensa P., Wieler L.H., Karch H., Reeves P.R., Maiden M.C., Ochman H. (2006). Sex and virulence in *Escherichia coli*: An evolutionary perspective. Mol. Microbiol..

[34] Chen L., Chen Z.L., Liu J.H., Zeng Z.L., Ma J.Y., Jiang H.X. (2007). Emergence of RmtB methylase-producing *Escherichia coli* and *Enterobacter cloacae* isolates from pigs in China. J. Antimicrob. Chemother..

[35] Carattoli A., Bertini A., Villa L., Falbo V., Hopkins K.L., Threlfall E.J. (2005). Identification of plasmids by PCR-based replicon typing. J. Microbiol. Methods.

[36] Johnson T., Bielak E., Fortini D., Hansen L., Hasman H., Debroy C., Nolan L., Carattoli A. (2012). Expansion of the IncX plasmid family for improved identification and typing of novel plasmids in drug-resistant Enterobacteriaceae. Plasmid.

[37] Chen L., Chavda K.D., Al Laham N., Melano R.G., Jacobs M.R., Bonomo R.A., Kreiswirth B.N. (2013). Complete nucleotide sequence of a *bla*_KPC_-harboring IncI2 plasmid and its dissemination in New Jersey and New York hospitals. Antimicrob. Agents Chemother..

[38] Larsen M., Cosentino S., Rasmussen S., Friis C., Hasman H., Marvig R., Jelsbak L., Sicheritz-Pontén T., Ussery D., Aarestrup F. (2012). Multilocus sequence typing of total-genome-sequenced bacteria. J. Clin. Microbiol..

[39] Alikhan N., Petty N., Ben Zakour N., Beatson S. (2011). BLAST Ring Image Generator (BRIG): Simple prokaryote genome comparisons. BMC Genom..

[40] Sullivan M., Petty N., Beatson S. (2011). Easyfig: A genome comparison visualizer. Bioinformatics.

[41] Cui C.Y., Chen C., Liu B.T., He Q., Wu X.T., Sun R.Y., Zhang Y., Cui Z.H., Guo W.Y., Jia Q.L. (2020). Co-occurrence of Plasmid-Mediated Tigecycline and Carbapenem Resistance in *Acinetobacter* spp. from Waterfowls and Their Neighboring Environment. Antimicrob. Agents Chemother..

[42] Bourafa N., Chaalal W., Bakour S., Lalaoui R., Boutefnouchet N., Diene S., Rolain J. (2018). Molecular characterization of carbapenem-resistant Gram-negative bacilli clinical isolates in Algeria. Infect. Drug Resist..

[43] Kanzari L., Ferjani S., Saidani M., Hamzaoui Z., Jendoubi A., Harbaoui S., Ferjani A., Rehaiem A., Boutiba Ben Boubaker I., Slim A. (2018). First report of extensively-drug-resistant *Proteus mirabilis* isolate carrying plasmid-mediated *bla*_NDM-1_ in a Tunisian intensive care unit. Int. J. Antimicrob. Agents.

[44] Aires-de-Sousa M., de la Rosa J.O., Goncalves M., Costa A., Nordmann P., Poirel L. (2020). Occurrence of NDM-1-producing *Morganella morganii* and *Proteus mirabilis* in a single patient in Portugal: Probable in vivo transfer by conjugation. J. Antimicrob. Chemother..

[45] Valentin T., Feierl G., Masoud-Landgraf L., Kohek P., Luxner J., Zarfel G. (2018). *Proteus mirabilis* harboring carbapenemase NDM-5 and ESBL VEB-6 detected in Austria. Diagn. Microbiol. Infect. Dis..

[46] Barbieri N., Pimenta R., de Melo D., Nolan L., de Souza M., Logue C. (2021). *mcr-1* Identified in Fecal *Escherichia coli* and Avian Pathogenic *E. coli* (APEC) From Brazil. Front. Microbiol..

[47] Lv Z., Shen Y., Liu W., Ye H., Liu D., Liu J., Fu Y., Peng C., Chen K., Deng X. (2022). Prevalence and risk factors of *mcr-1*-positive volunteers after colistin banning as animal growth promoter in China: A community-based case-control study. Clin. Microbiol. Infect..

[48] Zhang S., Huang Y., Yang G., Lei T., Chen M., Ye Q., Wang J., Gu Q., Wei X., Zhang J. (2021). High prevalence of multidrug-resistant *Escherichia coli* and first detection of IncHI2/IncX4-plasmid carrying *mcr-1 E. coli* in retail ready-to-eat foods in China. Int. J. Food Microbiol..

[49] Liu B., Li X., Zhang Q., Shan H., Zou M., Song F. (2019). Colistin-Resistant *mcr*-Positive Enterobacteriaceae in Fresh Vegetables, an Increasing Infectious Threat in China. Int. J. Antimicrob. Agents.

[50] Sun J., Zhang H., Liu Y.H., Feng Y. (2018). Towards Understanding MCR-like Colistin Resistance. Trends Microbiol..

[51] Kopotsa K., Sekyere J.O., Mbelle N.M. (2019). Plasmid evolution in carbapenemase-producing Enterobacteriaceae: A review. Ann. N. Y. Acad. Sci..

[52] Liu B., Guo Y., Liu N., Wang J., Li F., Yao L., Zhuo C. (2021). In silico Evolution and Comparative Genomic Analysis of IncX3 Plasmids Isolated From China Over Ten Years. Front. Microbiol..

[53] Xie X., Zhang J., Wang H.N., Lei C.W. (2021). Whole genome sequence of a New Delhi metallo-beta-lactamase 1-producing *Proteus mirabilis* isolate SNYG35 from broiler chicken in China. J. Glob. Antimicrob. Resist..

[54] Zhu X., Zhang Y., Shen Z., Xia L., Wang J., Zhao L., Wang K., Wang W., Hao Z., Liu Z. (2021). Characterization of NDM-1-Producing Carbapenemase in *Proteus mirabilis* among Broilers in China. Microorganisms.

[55] Toleman M.A., Bennett P.M., Walsh T.R. (2006). ISCR elements: Novel gene-capturing systems of the 21st century?. Microbiol. Mol. Biol. Rev..

[56] Li J., Lan R., Xiong Y., Ye C., Yuan M., Liu X., Chen X., Yu D., Liu B., Lin W. (2014). Sequential isolation in a patient of *Raoultella planticola* and *Escherichia coli* bearing a novel ISCR1 element carrying *bla*_NDM-1_. PLoS ONE.

