# Supplementary material for: Whole-Genome Analysis of blaNDM-Bearing Proteus mirabilis Isolates and mcr-1-Positive Escherichia coli Isolates Carrying blaNDM from the Same Fresh Vegetables in China
Source: Foods. 2023 Jan 20;12(3):492. doi: 10.3390/foods12030492 (PMC9913981; doi:10.3390/foods12030492)
Supplement: Supplementary file 1 [file foods-12-00492-s001.zip › foods-2097835-supplementary.pdf]

Table S1. Information about strains used for *Escherichia coli* evolutionary tree from NCBI database

Strains	GenBank assembly genome accession	Countries	Sources	*mcr-1*	bla_{NDM}
IHIT27704	GCA_001676985.1	Germany	Animal	+	
ICBEC3AM	GCA_001957205.1	Brazil	Environment	+	
O177:H21	GCA_001693635.1	Netherlands	Human	+	
SLK172	GCA_001936315.1	China	Human	+	
M4	GCA_002149955.1	China	Human	+	
ECO3347	GCA_002317635.1	China	Human	+	
CFSAN064036	GCA_003030025.1	Denmark	Human	+	
620	GCA_001892355.1	China	Human	+	
452	GCA_001891385.1	China	Human	+	
MDR_56	GCA_002009315.1	United States	Human	+	
ZJ3920	GCA_003076395.1	China	Human	+	
EcIB36	GCA_003248265.1	Bolivia	Human	+	
A20	GCA_003302575.1	China	Human	+	
A348	GCA_003303775.1	China	Human	+	
IHIT23707	GCA_003337065.1	Italy	Human	+	
PN101	GCA_003829975.1	Thailand	Human	+	
GBGD25	GCA_003859125.1	China	Human	+	
GBGD26	GCA_003860195.1	China	Human	+	
L935	GCA_004122195.1	China	Human	+	
CF10	GCA_003009875.1	China	Animal	+	
YH17178	GCA_002941225.1	China	Animal	+	
SWGE6	GCA_002930245.1	China	Animal	+	
I003p	GCA_009392935.1	China	Animal	+	
H2	GCA_015687395.1	China	Human	+	
18FS1-1	GCA_016124095.1	China	Animal	+	
2016FS061	GCA_019351155.1	China	Animal	+	
CJXMCRO520	GCA_019436665.1	China	Animal	+	
4862	GCA_020782775.1	China	Human	+	
ZJ1432	GCA_022546255.1	China	Human	+	
19ZY218	GCA_023600825.1	China	Human	+	
Tc-S1454	GCA_002283425.1	Colombia	Human	+	
G26	GCA_009823315.1	China	Human	+	
G25	GCA_009823355.1	China	Human	+	
50080	GCA_017593765.1	Lebanon	Animal	+	
20-AB01461	GCA_019448135.1	Germany	Animal	+	
UTA30	GCA_019492305.1	Ecuador	Environment	+	
AH25	GCA_013371725.1	China	Animal	+	
6409	GCA_000814145.2	Colombia	Human		+
CH613_eco	GCA_002133365.1	China	Human		+
BA13882	GCA_003004645.1	India	Human		+

ecoli019	GCA_900618295.1	Switzerland	Human	+
3R	GCA_011067085.1	China	Animal	+
MS6194	GCA_013363225.1	India	Human	+
MS6204	GCA_013363395.1	India	Human	+
elppa8	GCA_020827595.1	China	Human	+
MS6203	GCA_013363335.1	United Kingdom	Human	+
ME2L-20-113	GCA_019834435.1	United States	Environment	+
HD6415	GCA_020162075.2	China	Environment	+
E-TJ2-1	GCA_021342175.1	China	Human	+
E-TJ9-1	GCA_021342235.1	China	Human	+
E-TJ6-1	GCA_021342345.1	China	Human	+
E-TJ4-2	GCA_021342395.1	China	Human	+
EC5502	GCA_022453585.1	China	Human	+
NDM5	GCA_020149625.1	United States	Human	+
51008369SK1	GCA_003254065.1	Switzerland	Animal	+
ECONIH6	GCA_002903125.1	United States	Human	+
CRE1493	GCA_002860105.1	China	Human	+
